# Characterization of an aspartate aminotransferase encoded by YPO0623 with frequent nonsense mutations in *Yersinia pestis*

**DOI:** 10.3389/fcimb.2023.1288371

**Published:** 2023-11-28

**Authors:** Junyan Jin, Liting Xiao, Yarong Wu, Zhulin Sun, Ziyao Xiong, Yanbing Li, Yanting Zhao, Wenwu Yao, Leiming Shen, Yiming Cui, Yafang Tan, Yanping Han, Zongmin Du, Yujun Cui, Ruifu Yang, Kai Song, Yajun Song

**Affiliations:** ^1^ State Key Laboratory of Pathogen and Biosecurity, Beijing Institute of Microbiology and Epidemiology, Beijing, China; ^2^ School of Basic Medical Sciences, Anhui Medical University, Hefei, China; ^3^ College of Life Sciences, Fujian Agriculture and Forestry University, Fuzhou, China; ^4^ Department of Laboratory Medicine, Xiangya Hospital of Central South University, Changsha, China; ^5^ College of Horticulture and Plant Protection, Inner Mongolia Agricultural University, Hohhot, China; ^6^ Department of Microbiology and Department of Infectious Diseases, Zhejiang Provincial Center for Disease Control and Prevention, Hangzhou, China

**Keywords:** *Yersinia pestis*, YPO0623, aspartate aminotransferase, T6SS, genome decay

## Abstract

*Yersinia pestis*, the causative agent of plague, is a genetically monomorphic bacterial pathogen that evolved from *Yersinia pseudotuberculosis* approximately 7,400 years ago. We observed unusually frequent mutations in *Y. pestis* YPO0623, mostly resulting in protein translation termination, which implies a strong natural selection. These mutations were found in all phylogenetic lineages of *Y. pestis*, and there was no apparent pattern in the spatial distribution of the mutant strains. Based on these findings, we aimed to investigate the biological function of YPO0623 and the reasons for its frequent mutation in *Y. pestis*. Our *in vitro* and *in vivo* assays revealed that the deletion of YPO0623 enhanced the growth of *Y. pestis* in nutrient-rich environments and led to increased tolerance to heat and cold shocks. With RNA-seq analysis, we also discovered that the deletion of YPO0623 resulted in the upregulation of genes associated with the type VI secretion system (T6SS) at 26°C, which probably plays a crucial role in the response of *Y. pestis* to environment fluctuations. Furthermore, bioinformatic analysis showed that YPO0623 has high homology with a PLP-dependent aspartate aminotransferase in *Salmonella enterica*, and the enzyme activity assays confirmed its aspartate aminotransferase activity. However, the enzyme activity of YPO0623 was significantly lower than that in other bacteria. These observations provide some insights into the underlying reasons for the high-frequency nonsense mutations in YPO0623, and further investigations are needed to determine the exact mechanism.

## Introduction

*Yersinia pestis* is the pathogenic bacteria that causes plague, a naturally occurring epidemic disease (Mordechai et al., 2019; Yang et al., 2023). *Y. pestis* has been proven to evolve from its ancestor *Yersinia pseudotuberculosis* around 7400 years ago with limited genomic polymorphisms (Rascovan et al., 2019; Susat et al., 2021). We performed a systematic analysis of public *Y. pestis* genomes to investigate the fundamental characteristics of SNP and Indel variation throughout the entire genome. Interestingly, we observed a significantly higher mutation frequency in YPO0623 (CO92 genome, GCF_000009065.1) compared to the average frequency across the entire *Y. pestis* genome, which is estimated to range from 3.1×10^−9^ to 1.3×10^−7^ per site per year (Cui et al., 2013).

To trace the associations between the evolution of *Y. pestis* and the polymorphism in YPO0623, we performed the expression of YPO0623 and assessed its enzymatic activity. Our results revealed that the YPO0623 protein displayed aspartate aminotransferase activity, but significantly lower than that observed in other bacterial species like *Escherichia coli*. Furthermore, we performed a knock-out experiment targeting YPO0623 in *Y. pestis* Microtus strain 201 and observed that the loss of this gene provided certain fitness advantages to the bacteria, particularly in terms of growth and under various stressful conditions such as cold shock and heat shock. Based on these findings, we hypothesize that YPO0623 functions as an aspartate aminotransferase in *Y*. *pestis*, albeit with significantly lower enzyme activity compared to *aspC* (Nikaido and Vaara, 1985). Thus, mutations that result in the pseudogenization of YPO0623 render fitness advantages during its natural life cycle stages.

## Results

### Polymorphism of *Y. pestis* YPO0623

**Figure 1:**
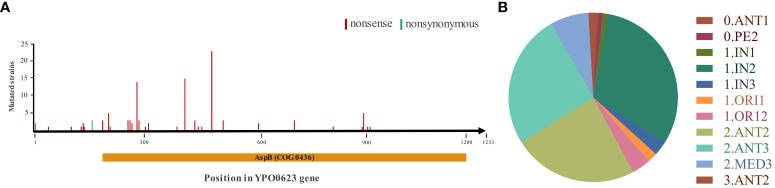
Distribution of YPO0623 mutations in 109 *Y. pestis* strains. **(A)** Mutation locations of YPO0623 and the numbers of strains with individual mutations in 109 strains. Nonsense, nonsense mutation; nonsynonymous, nonsynonymous mutation. AspB (COG0436), conserved domain identified by CD-Search in the Conserved Domain Database in NCBI. **(B)** Distribution of 109 strains with YPO0623 mutations across the phylogenetic lineages of *Y. pestis*.

To investigate the characteristics of single nucleotide polymorphisms (SNPs) and Indel variations of YPO0623, we performed a comparative analysis of all published genomic data to date (3318) for *Y. pestis*. Specifically, 109 *Y. pestis* strains exhibited a single mutation occurring in different positions of the YPO0623 gene (**Figure 1A**). The mutations in 104 strains cause premature termination of protein translation. Additionally, three out of the five remaining mutations result in nonsynonymous mutations (D52A), while the other two lead to nonsynonymous mutations in the start codon (M1I). This finding suggests that natural selection might play a significant role in shaping the *Y. pestis* YPO0623 gene, and the loss of YPO0623 protein may provide adaptive advantages for the organism. Moreover, these mutations are not confined to specific lineages but rather distributed across multiple evolutionary lineages (**Figure 1B**), indicating that the observed polymorphisms might be driven by certain stresses.

### YPO0623 enzyme activity verification

**Figure 2:**
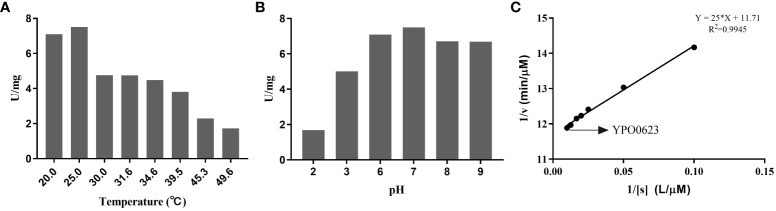
Determination of the enzyme activity of YPO0623. **(A)** Determination of optimum temperature of YPO0623 enzyme activity. The activity was examined at various temperatures ranging from 20.0°C to 50.0°C in reaction system (pH 7.0) for 3 h **(B)** Determination of optimum pH of YPO0623 enzyme activity. The activity was examined at various pH ranging from 2.0 to 9.0 in reaction system (25.0°C) for 3 h **(C)** 1/v-1/[s] double reciprocal plot for Km. The activity was examined in reaction system (25.0°C, pH=7.0).

The YPO0623 protein displayed a high degree of similarity with the pyridoxal phosphate (PLP)-dependent aspartate aminotransferase of *Salmonella enterica*, exhibiting 99% homology and “CD-Search” analysis (https://www.ncbi.nlm.nih.gov/cdd) revealed an AspB (COG0436) conserved domain in YPO0623 (**Figure 1A**). BL21 containing pET-28(a+)-YPO0623 was induced by 1 mM IPTG, and the eluate with Wash Buffer containing different concentrations of imidazole were subjected to SDS-PAGE. The results showed that the target protein was around 50 kDa and was obtained under 250 mM imidazole elution (**Supplementary Figure 1**). Using the NADH-Na_2_ standard curve obtained at various pH levels, we performed enzyme activity measurements of the YPO0623 protein across a range of temperatures and pH values (**Supplementary Figure 2**). Enzyme activity assays were performed between 20.0°C and 49.6°C, and the results showed that the highest activity was achieved at 25.0°C (**Figure 2A**), reaching 7.51 U/mg. Additionally, measurements were taken at 25°Cwith varying pH values ranging from 2.0 to 9.0, and the optimal pH for the enzyme activity was determined to be 7.0 (**Figure 2B**), at which the activity reached 7.49 U/mg. A range of substrate concentrations was utilized under the optimal conditions of pH 7.0 and 25°C, and double reciprocal plots were generated (**Figure 2C**). From these plots, we determined the Km value of the enzyme to be 2.13 mol/L, Kcat to be 0.03 s, and Vmax to be 0.09 mol/min. Notably, the aspartate aminotransferase activity of the YPO0623 protein in *Y. pestis* was considerably lower compared to that in other bacteria (**Table 1**), indicating that the low enzyme activity of YPO0623 and the presence of alternative metabolic pathways might contribute to its pseudogenization in some *Y. pestis* strains.

**Table 1:** Km values of aspartate aminotransferase from different bacteria.

Bacteria	Gene/Protein name	Km (μM)	References
*Yersinia pestis*	YPO0623	2.13	This study
*Pseudomonas striata*	NA	450	(Yagi et al., 1976)
*Escherichia coli*	*aspC*	450	(Powell and Morrison, 1978)
*Geobacillus thermopakistaniensis*	AST_Gt_	1500	(Gharib et al., 2020)
*Thermus thermophilus* HB8	AspAT1	1800	(Okamoto et al., 1996)
*Streptomyces fradiae*	ASAT	2700	(Lee and Lee, 1993)
*thermophilic Bacillus species*	AspAT	3000	(Sung et al., 1990)
*Phormidium lapideum*	AspAT	5000	(Kim et al., 2003)
*Haloferax mediterranei*	AspAT	12600	(Muriana et al., 1991)
*Pseudoalteromonas haloplanktis* TAC 125	PhAspAT	64700	(Birolo et al., 2000)

### Construction of the null mutant of YPO0623 and assessment of its growths

**Figure 3:**
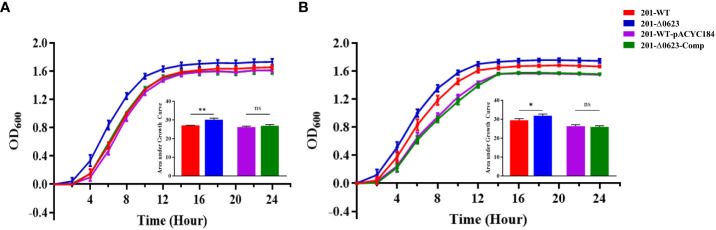
Growth curves determination of 201-Δ0623 and 201-WT. **(A)** Growth curve of 201-WT, 201-Δ0623, 201-WT-pYCAC184, and 201-Δ0623-Comp at 26°C in LB. **(B)** Growth curve of 201-WT,201-Δ0623, 201-WT-pYCAC184, and 201-Δ0623-Comp at 37°C in LB. The under the growth curves is the area under the curves of the growth curves. The bar graph presented below the growth curves accurately illustrates the cumulative areas under the curves and is applied to statistical analysis. *,p < 0.05; **,p < 0.01; ns, non-significant.

To investigate the functional role of YPO0623 in *Y. pestis*, we employed the suicide vector pDS132 to construct the null mutant named 201-Δ0623 based on *Y. pestis* the biovar Microtus strain 201. Corresponding complemented strains were constructed following the procedures outlined in the Materials and Methods section. The mutants were subsequently verified through PCR and sequencing. The result of growth curves *in vitro* for 201-Δ0623 and wild-type strain 201 (known as 201-WT) in LB medium showed that the growth rate and the maximum growth value of 201-Δ0623 were significantly higher than those of 201-WT at both 26°C and 37°C (**Figure 3**). These results suggest that the deletion or inactivation of YPO0623 may lead to more efficient growth and reproduction of *Y. pestis* in the medium.

### Deletion of YPO0623 does not affect the biofilm formation of *Y*. *pestis*

**Figure 4:**
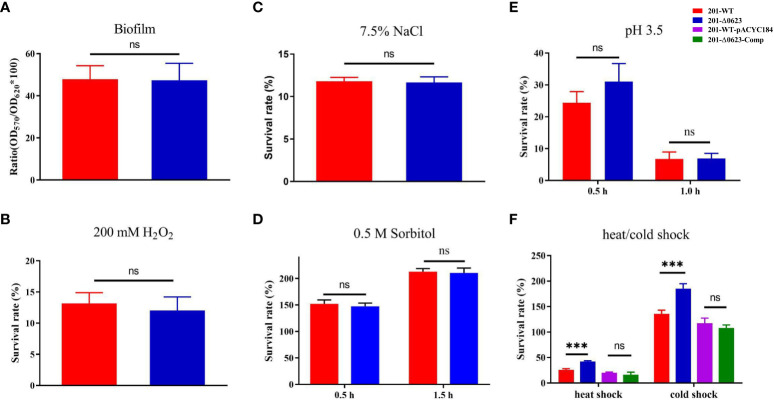
Comparison of the biofilm formation and viabilities of 201-WT and 201-Δ0623 in various conditions. **(A)** Semi-quantitative determination of biofilm formation of two strains using crystal violet. Survival rate of 201-WT and 201-Δ0623 exposed to 200 mM H_2_O_2_ for 15 minutes **(B)**; exposed to 7.5% NaCl for 1 hour **(C)**; exposed to 0.5 M C_6_H_14_O_6_ for 0.5 or 1.5 hours **(D)**; exposed to acid stimulation (pH=3.5) for 0.5 or 1.0 hours **(E)**; exposed to cold shock (4°C for 24 hours) or heat shock (50°C for 30 minutes) **(F)**. Data was analyzed by *t*-test. ***,p < 0.001; ns, non-significant.

Biofilm plays a crucial role in the transmission of bacteria through fleas. To analyze the influence of YPO0623 gene knockout on *Y. pestis* biofilm formation, a semi-quantitative assessment was performed using 0.1% crystal violet on both 201-WT and 201-Δ0623. Our findings indicate that there is no significant difference in biofilm formation between the two strains (**Figure 4A**). Therefore, it can be concluded that the knockout of the YPO0623 does not impact the biofilm formation of *Y*. *pestis*.

### Differences in survival of 201-WT and 201-Δ0623 under various stress conditions

Bacteria must swiftly respond and adapt themselves to various challenging environments to ensure their survival. To simulate the environmental conditions that *Y. pestis* may encounter in nature, we set certain stress environments including high salinity, high permeability, low pH levels, exposure to reactive oxygen species, cold shock, and heat shock. After being exposed to 200 mM H_2_O_2_ stress for 15 minutes, there was no significant difference in survival rate between the 201-Δ0623 and 201-WT strains (**Figure 4B**). Similarly, when subjected to 1 hour of high salt stress at 7.5% NaCl, both strains had similar survival rates (**Figure 4C**). Additionally, when subjected to 0.5 M sorbitol hypertonic stress for 0.5 hours and 1.5 hours, or to acid stimulation (pH=3.5) for 0.5 hours or 1 hour, no significant differences in survival rates were observed between the 201-WT and 201-Δ0623 strains (**Figures 4D, E**). However, when exposed to a temperature of 4°C for 24 hours and 50°C for 30 minutes, the 201-Δ0623 demonstrated significantly higher survival rates in comparison to the 201-WT (**Figure 4F**). This suggests that the deletion of the YPO0623 gene enhances the ability of *Y. pestis* to withstand external temperature fluctuations like cold or heat shocks.

### Deletion of YPO0623 does not affect the virulence on BALB/c mice

**Figure 5:**
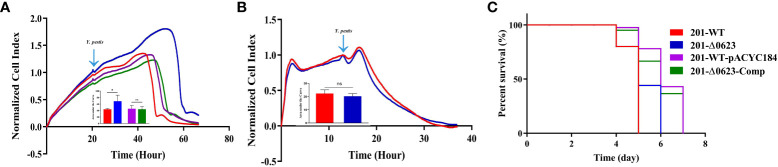
Determination of the cytotoxicity and virulence on mice of the strains. Results of real-time cell analysis assay of **(A)** HeLa cells infected with 201-WT, 201-Δ0623, 201-WT-pYCAC184, and 201-Δ0623-Comp and **(B)** Raw264.7 cells infected with 201-WT and 201-Δ0623. The bar graph presented below the curves accurately illustrates the cumulative areas under the curves and is applied to statistical analysis. *,p < 0.05; ns, non-significant. **(C)** Survival curves of mice challenged by 201-WT and 201-Δ0623.

We further assessed whether the loss of YPO0623 affects the cytotoxicity on cells or the virulence on mice of *Y. pestis*. RTCA (Real-time Cell Analysis) is capable of assessing infected cells that are placed onto a gold matrix by measuring the cell index (CI), which reflects cellular changes including cell adhesion and cell number (Kho et al., 2015; Roshan Moniri et al., 2015; Cetin and Topcul, 2019). Our data from 10 to 55 hours post-infection (or 20 to 65 hours in the RTCA assay) revealed significantly lower CI readouts in HeLa cells infected with the 201-WT strain compared to those infected with the 201-Δ0623 strain (**Figure 5A**). However, no significant differences were observed in CI readouts in Raw246.7 cells infected with either the 201-WT or the 201-Δ0623 strain (**Figure 5B**). RTCA results indicated that the loss of YPO0623 in *Y. pestis* reduced its cytotoxicity on epithelioid cells, but made no differences in the cytotoxicity on macrophages.

Furthermore, our results demonstrated no significant difference in virulence between the two strains in BALB/c mice (**Figure 5C**). This implies that the knockout of the YPO0623 gene had no impact on the virulence of *Y. pestis* in BALB/c mice. Specifically, the mortality rate of mice infected with 201-WT strain was 100% by Day 5, while those infected with 201-Δ0623 strain had a 100% mortality rate by Day 6.

### Comparative transcriptomics of 201-WT and 201-Δ0623 strains

To better investigate the influence of YPO0623 gene knockout on *Y. pestis*, we performed transcriptomic analysis on strains 201-WT and 201-Δ0623 cultivated at temperatures of 26°C or 37°C. Under the 26°C culture condition, we observed that 201-Δ0623 exhibited 269 up-regulated genes and 271 down-regulated genes compared to 201-WT (**Supplementary Figure 3A**). Under the 37°C culture condition, 201-Δ0623 displayed 936 up-regulated genes and 959 down-regulated genes relative to 201-WT (**Supplementary Figure 3B**). Furthermore, KEGG enrichment analysis shows that many genes associated to cellular processes or metabolic pathways up-regulated in 201-Δ0623 compared to 201-WT, including carbon metabolism, histidine metabolism, and oxidative phosphorylation, etc. While, under 26°C, expression of genes involved in biosynthesis of amino acids, cysteine and methionine metabolism, and phosphotransferase system down-regulated in Δ0623 compared to 201-WT (**Supplementary Figure 4**). To validate the RNA-seq data, we performed qRT-PCR analysis of selected genes using the same RNA samples used for the construction of the RNA-seq libraries. Notably, we obtained high correlation coefficients (R^2^) between the RNA-seq and qRT-PCR results (>0.90) for the RNA samples obtained at both 26°C and 37°C (**Supplementary Figure 3C**). Interestingly, our RNA-seq analysis revealed that genes associated with the type VI secretion system (T6SS), including *tssB, tssC, tssE, tssF, tssH, tssG, tssJ*, and *tssK*, were upregulated in 201-Δ0623 compared to strain 201 at 26°C, as well as to the complementary strain 201-Δ0623-Comp (**Table 2**).

**Table 2:** The genes of T6SS were upregulated in 201-Δ0623 in comparison with 201-WT.

gene_id	gene name	log_2_FC (Δ0623 vs WT)		log_2_FC (Δ0623 vs Comp)	gene description
		RNA-seq	qRT-PCR	qRT-PCR	
YP_RS15960	*tssE*	1.27	1.67	1.55	type VI secretion system baseplate subunit TssE
YP_RS15965	*tssC*	1.08	1.38	1.35	type VI secretion system contractile sheath large subunit
YP_RS15970	*tssB*	1.18	1.09	1.04	type VI secretion system contractile sheath small subunit
YP_RS19140	*tssK*	1.63	1.89	1.83	type VI secretion system baseplate subunit TssK
YP_RS19145	*tssJ*	1.59	1.77	2.67	type VI secretion system lipoprotein TssJ
YP_RS19175	*tssH*	1.51	1.32	1.30	type VI secretion system ATPase TssH
YP_RS19170	*vgrG*	1.55	*-*	–	type VI secretion system tip protein VgrG
YP_RS19180	*tssG*	1.59	1.76	1.65	type VI secretion system baseplate subunit TssG
YP_RS19185	*tssF*	1.71	1.98	1.92	type VI secretion system baseplate subunit TssF
YP_RS19200	*tssC*	1.57	1.72	1.65	type VI secretion system contractile sheath large subunit
YP_RS19205	*tssB*	1.65	1.67	1.71	type VI secretion system contractile sheath small subunit

## Materials and methods

### Bacterial strains and culture conditions

A list of bacterial strains and plasmids used in this study can be found in **Supplementary Table 1**. The strain used in this research was the *Y. pestis* biovar Microtus strain 201, a human-avirulent strain while lethal in mice. Strain 201 was isolated from the rodent *Microtus brandti* in Inner Mongolia, China. This strain carried an intact YPO0623 gene and possessed a genome sequence consistent with that of the *Y. pestis* strain 91001 (Song et al., 2004). The strains were recovered from frozen stocks, and the second-passage cultures were employed for subsequent investigations. The strains were cultured in Lysogeny broth (LB) broth (pH 7.4) or on LB agar plates at temperatures of 26°C or 37°C for different assays. When necessary, the media were supplemented with 34 or 6.8 μg/mL of chloramphenicol (Cm) or 50 μg/mL of kanamycin (Kan).

### DNA preparation

Plasmids and genomic DNA were extracted separately following the manufacturer’s instructions, utilizing the QIAprep Spin Miniprep Kit (Qiagen, Germany) for plasmids and the QIAamp DNA Mini Kit (Qiagen, Germany) for genomic DNA. Polymerase chain reaction (PCR) was performed using the Bio-Rad T100TM thermal cycler and the SanPfu PCR Mix (Sangon, China). The PCR consisted of 30 cycles, involving denaturation at 95°C for 30 seconds, annealing at the temperature determined by the primers used for 30 seconds, and extension at 72°C for a duration appropriate to the length of the target gene. Last, an additional extension step was executed at 72°C for 5 minutes.

### Construction and identification of the mutant strains, complementation strains

To generate the upstream and downstream homology arms of YPO0623 from strain 201, we utilized the Pre0623-F/R and Post0623-F/R primer pairs. The resulting amplification products were purified using a QIAquick PCR Purification Kit (Qiagen, Germany). Sph I and Sac I enzymes (LMAI bio, China) were employed to digest plasmid pDS132. The homology arms and digested pDS132 were ligated using 2×Seamless Cloning Mix (Biomed, China) at 50°C for 15 min, followed by introduction into competent *E. coli* S17-1λpir to generate *E. coli* S17-1 λpir-pDS132-YPO0623del.

Both strain 201-WT and *E. coli* S17-1 λpir-pDS132-YPO0623del were cultured in LB medium until reaching an OD_620_ or OD_600_ of 1.0. Following centrifugation of 1.5 mL of *E. coli* S17-1 λpir-pDS132-YPO0623del and 100 μL of strain 201 cultures at 2000 g for 5 min, the cells were resuspended in 50 μL of LB medium. The cells were then thoroughly mixed and pipetted onto a filter paper (4.5 μm) attached to an LB plate. The plate was incubated overnight at 26°C. Yersinia Selective Agar Base (Oxoid, UK) plate with 6.8 μg/mL Chloromycetin was used for the isolation and culture of *Y. pestis*. Single colonies were selected after incubating at 26°C for 4-5 days and inoculated in LB medium. The culture was then coated on LB plates containing 7% sucrose for screening and then incubated at 26°C for 3 days. PCR verification was performed using Inter 0623-F/R primers, and the amplification results were confirmed by DNA sequencing.

To generate the complemented strains, we cloned a PCR-amplified DNA fragment of the YPO0623 coding sequences into pACYC184 at Hind III and BamH I sites (LMAI bio, China). The ligation reaction was performed as described above. After verifying the DNA sequence, we transferred the recombinant plasmid into strain 201-Δ0623. **Supplementary Table 2** provides detailed information on the primer combinations employed in this process.

### Construction of the protein expression vector

The YPO0623 PCR-amplified fragment was cloned into pET-28 (a+) at Hind III and BamH I sites (LMAI bio, China). And the resulting plasmids were then transferred into BL21 (ED3) (Sangon, China). **Supplementary Table 2** provides detailed information on the primer sequences employed in this process.

### YPO0623 protein expression and purification

BL21 (DE3) colonies containing the pET-28a (+)-YPO0623 plasmid were selected and inoculated into LB liquid medium supplemented with 50 μg/mL kanamycin. The cultures were shaking at 37°Cuntil reaching an optical density at 600 nm (OD_600_) of approximately 1.0. Subsequently, the entire culture was transferred into 800 mL of fresh LB liquid medium supplemented with 50 μg/mL kanamycin and further shaking at 37°C. When the OD_600_ reached approximately 0.6, the culture was placed on ice for 5 minutes, and then 800 μL of IPTG (1 mM) was added. The culture was shaking overnight at 18°C.

The bacterial cells were harvested by centrifugation at 9000 g for 10 minutes at 4°C. The pellet was washed using PBS buffer twice and resuspended 30 mL of lysis buffer (containing 500 mM NaCl, 350 mM NaH_2_PO_3_, 10 mM imidazole, pH=8.5) supplemented with 300 μL of protease inhibitor. The samples were then subjected to ultrasonication on ice for 30 minutes, followed by centrifugation at 8000 g for 30 minutes at 10°C. A 3 mL Ni-NTA affinity chromatography column was loaded with the supernatant. Elution was performed using different concentrations of imidazole solution, and each eluate was analyzed by SDS-PAGE. The fractions containing the target protein were collected and subjected to desalting using a G25 fast desalting column (Bersee, China).

### Enzyme activity verification

The reaction system was prepared as follows: 1.5 μM of pyridoxal 5’-phosphate (PLP), 10 mM of Tris-HCl, 200 μM of aspartic acid, 20 μM of NADH-Na_2_, 200 U of malate dehydrogenase, and 1 mM of α-ketoglutaric acid (Solarbio, China) were added to the mixture following the published protocol (Kim et al., 2003). The NADH-Na_2_ standard curves under different pH conditions were established. To investigate the effect of temperature on enzyme activity, the reaction system was incubated at different temperatures (20-50°C) and pH=7.0 for 3 hours, after which the enzyme activity was measured. To study the impact of pH on the enzyme reaction, the reaction system was incubated at different pH values (2.0-9.0) and 25°C for 3 hours, and the enzyme activity was measured. Substrate solutions with varying concentrations (pH 7.0) were prepared and reacted with the enzyme solution at 25°C. Double reciprocal plotting was utilized to determine the values of Km, Kcat, and Vmax. The experiment was independently replicated three times, and the results were expressed as mean ± standard deviation (n = 3).

### Growth curve determination

*Y. pestis* strains were cultured in LB medium or TMH medium at either 26°C or 37°C until reaching an approximate OD_620_ of 1.0, corresponding to around 2 × 10^8^ CFU/mL. Following that, bacterial cultures were diluted 1:20 in 20 mL of fresh LB or TMH medium in a 50 mL Erlenmeyer flask and incubated at the same temperature as before with shaking at 220 r/min. The experiment was performed using three independent biological replicates, and the results were reported as the mean and standard deviation of those three trials.

### Determining biofilm formation of the mutant

To fully activate two glycerol bacteria strains, 201-WT and 201-Δ0623, they were subcultured until reaching an OD_620_ of approximately 1.0. Subsequently, they were centrifuged at 2000 g for 5 minutes, suspended in LB liquid culture, and diluted 20 times. The bacterial suspensions were then placed in a 24-well plate and shaken at 26°C and 200 r/min for 24 hours. The OD_620_ and biofilm formation were measured by removing the bacterial liquid from the 24-well plate. After washing twice with ddH_2_O, the biofilm was fixed at 80°C for 15 minutes in a hybridization furnace. Then, 3 mL of 0.1% crystal violet was added dropwise for staining for 15 minutes, followed by washing twice with ddH_2_O. Subsequently, 3 mL of anhydrous ethanol was added to each well. The plates were left at room temperature for 2 hours before determining the OD_570_ after five-fold dilution with anhydrous ethanol. The relative biofilm formation was calculated as 100 × OD_570_/OD_620_. This experiment was performed using three independent biological replicates, and the results were presented as the mean ± standard deviation (n=3).

### Determining the ability of the mutant to survive in different environments

Two glycerol-producing bacteria, 201-WT and 201-Δ0623, were fully activated, subcultured until reaching an OD_620_ of approximately 1.0 then centrifuged at 2000 g for 5 minutes. The bacterial pellets were re-suspended in PBS buffer containing 200 mM H_2_O_2_. The suspensions were diluted ten-fold and subjected to shaking culture at 26°C and 200 r/min for 15 minutes. The concentration of bacterial cells was determined by plating them onto Hottinger’s agar plates.

The same method described above was employed to determine the survival rate of the mutant after 2 hours of stimulation with 7.5% NaCl, to evaluate the bacterial survival rate after 0.5 hours or 1.5 hours of stimulation with 0.5 M sorbitol, and to determine the survival rate of the mutant after 0.5 hours and 1.0 hour of stimulation at pH 3.5.

Similarly, to determine the survival rate of *Y. pestis* after exposure to 24 hours at 4°C or 30 minutes at 50°C, the same method as described earlier was employed. This experiment was performed with three independent biological replicates, and the results were expressed as the mean ± standard deviation (n=3).

### Real-time cell analysis assay

HeLa or Raw264.7 cells were cultured in Dulbecco’s Modified Eagle’s Medium (DMEM, Solarbio, China) supplemented with 10% fetal bovine serum (FBS) and maintained at 37°C in a 5% CO_2_ incubator. To establish a baseline for the assay, 150 µL of DMEM with 10% FBS was added to each well of an E-plate attached to the RTCA iCELLigence system (ACEA Biosciences, USA), and the plate was maintained at 37°C in a 5% CO_2_ incubator. The concentration of HeLa cells was adjusted to 5×10^3^ cells/mL, while Raw264.7 cells were adjusted to 1×10^4^ cells/mL before pipetting into each well of the E-plate. After transferring the E-plate containing the cells to the RTCA iCELLigence system under the same conditions, it was incubated at 37°C for 30 minutes in a 5% CO_2_ incubator to establish a stable baseline. *Y. pestis* cells were collected and resuspended in sterile phosphate-buffered saline (PBS). HeLa cells or Raw264.7 cells were then infected with *Y. pestis* cells at a multiplicity of infection (MOI) of 5. The cell index (CI) was recorded every two minutes using the RTCA iCELLigence system during an incubation period of up to 10 hours.

### Mouse infection

*Y. pestis* was cultured using the previously mentioned method to achieve a concentration of approximately 2 × 10^8^ CFU/mL. The bacterial colonies were diluted in sterile PBS to the desired cell density, followed by centrifugation at 2000 g for 5 minutes and subsequent washing steps twice. The bacterial concentration was determined by plating on Hottinger’s agar dishes. Female BABL/c mice, aged 6 to 8 weeks, were procured from Beijing Vital River Laboratory Animal Technology Co. Ltd. Subcutaneous injection of 100 µL of cultures, appropriately diluted with PBS, was administered to the mice for infection with the 201-WT and mutant strains. All animals used in the experiment were handled in accordance with Chinese guidelines for the welfare and ethics of laboratory animals. Continuous mortality records were kept, and the log-rank (Mantel-Cox) test was employed to analyze the data, with statistical significance set at P<0.05.

### RNA isolation, sequencing, and data analysis

Three independent biological replicates of *Y. pestis* strain 201-WT and the 201-Δ0623 mutant were cultured in LB broth at temperatures of 26°C or 37°C to achieve a bacterial concentration of approximately 2 × 10^8^ CFU/mL. Total RNA was extracted using the RNAprep Pure Cell/Bacteria kit (Tiangen, China) and utilized for deep sequencing to generate a cDNA library. The ratio of transcript levels between the 201-WT and 201-Δ0623 groups was calculated as the logarithm to the base 2, based on fragments per kilobase of transcript per million mapped reads. A minimum fold change of two was employed to assess the differential expression of genes.

### Quantitative reverse transcription PCR analysis

The RNA samples intended for sequencing were subjected to reverse transcription using the TSINGKE TSK322S SynScriptTM III cDNA Synthesis Mix (Tsingke Biotechnology Co., Ltd., China) to generate cDNA templates. The accuracy of the RNA-seq results was confirmed through qRT-PCR. For qRT-PCR experiments, the 2T5 Fast qPCR Mix (SYBR Green I) was employed, and the Bio-RAD CFX Opus 96 system was utilized for the study. Linear regression analysis was employed to compare the qRT-PCR results with the sequencing data. Primer pairs were designed using primer design tools to generate amplicons with lengths ranging from 100 to 300 bp. Specific details regarding the primer combinations used can be found in **Supplementary Table 2**.

### Statistical analysis

The mean and standard deviation for each of the three experimental groups were determined based on three independent experiments. T-test was employed for data comparison, provided they satisfied the assumptions of normality and homogeneity of variance. When the data did not follow a normal distribution, the nonparametric test was performed. The survivorship curve was analyzed using the Mantel-Cox test for log-rank. The significance levels were represented as follows: *, p < 0.05; **, p < 0.01; and ***, p < 0.001.

## Discussion

This study provides the evidence that *Y. pestis* YPO0623 is an aspartate aminotransferase that relies on pyridoxal-5’-phosphate PLP as a cofactor. The optimal reaction temperature and pH were determined to be 25°C and 7.0, respectively, aligning with the optimal growth conditions for *Y. pestis*. The enzyme displayed a Km value of 2.13 μM/L, a Kcat value of 0.03/s, and a Vm value of 0.09 μM/min. Notably, the Km value of *Y. pestis* YPO0623 was significantly lower compared to the aspartate aminotransferase in other bacteria, indicating a relatively low enzymatic activity of this gene. Notably, the *aspC* gene in *Y. pestis* also functions as an aspartate aminotransferase (Song et al., 2004), suggesting the redundancy of this role in *Y. pestis*.

Upon examining publicly available *Y. pestis* genomes, we discovered frequent mutations in YPO0623 that resulted in the premature termination of protein synthesis. To investigate whether the loss of YPO0623 could confer *Y. pestis* any fitness advantages, we first evaluated the growth of the mutant 201-Δ0623 in LB liquid medium. Our findings revealed that the growth rate of 201-Δ0623 was notably higher than that of 201-WT at both 26°C and 37°C, indicating that the loss of YPO0623 expedited the growth and reproduction of *Y. pestis* in nutritionally favorable environments. Furthermore, we examined the effect of the YPO0623 deletion on the viability of *Y. pestis* under different stressful conditions. We observed that 201-Δ0623 had a significantly higher survival rate than 201-WT when exposed to cold shock or heat shock. This implies that the lack of YPO0623 provides *Y. pestis* with increased resilience in situations involving drastic temperature changes. In other words, the inactivation of YPO0623 could enhance the adaptability of *Y. pestis* to environments with prolonged cold or high temperatures, potentially improving its chances of survival.

RNA-seq data revealed that deletion of the YPO0623 gene resulted in the up-regulation of T6SS-related genes in *Y. pestis* at 26°C, most of which belonged to the T6SS-A gene cluster specific to *Y. pestis*. Phylogenetic analysis of T6SSs demonstrated a close genetic distance and similar genomic structure between the T6SS-A cluster of *Y. pestis* and the T6SS-4 cluster of *Y. pseudotuberculosis*(Yang et al., 2018). T6SS-4 is a temperature-controlled gene cluster identified in *Y. pseudotuberculosis* and is tightly regulated by temperature, growth stage, and an AHL-dependent quorum sensing system (Pieper et al., 2009; Yang et al., 2018). This gene cluster plays a significant role in resistance to environmental stress, suggesting that T6SS-A in *Y. pestis* likely is crucial for environmental adaptation. The regulation of T6SS-related gene expression may be associated with the bacterium’s adaptability and enhanced T6SS expression can aid *Y. pestis* in better adapting to its ambient environment and competing with other bacteria to gain survival advantages.

By screening a recently published *Y. pestis* multi-omics online database (https://yersiniomics.pasteur.fr/) (Le-Bury et al., 2023), we discovered that out of the 78 available transcriptomic datasets of *Y. pestis*, the transcriptional level of YPO0623 exhibited significant changes in just 25 experiments (up-regulated in 12 and down-regulated in 13), while remain stable in the rest 53 tested conditions. This suggests that YPO0623 may only respond to limited niche changes and might be dispensable for *Y. pestis* in certain cases. Therefore, it is plausible that the loss of YPO0623 gene may have neglectable effects under certain circumstances while fitness advantages under other circumstances in *Y. pestis*.

The process of genome decay normally occurs when certain genes or gene clusters are no longer necessary for the survival of microorganisms, or when the microorganisms adapt to new environments (Couce et al., 2017). This process results in the inactivation and loss of genes, which can streamline the genome and optimize microbial metabolism (Cerdeno-Tarraga et al., 2004). Genomic decay is a common phenomenon in the evolution of bacterial pathogens, enabling them to adapt to new environments more efficiently. The genome of *Y. pestis* is around 4.65 Mb and contains roughly 4,000 genes. Notably, approximately 10% of genes in the *Y. pestis* genome have been lost in comparison to the genome of its ancestor *Y. pseudotuberculosis *(Parkhill et al., 2001; Chain et al., 2004). The accumulation of pseudogenes may facilitate the speciation and microevolution of *Y. pestis* (Tong et al., 2005). For example, *yadA* and *inv* are essential for the intestinal pathogen *Y. pseudotuberculosis* to adhere to the host’s intestinal surface and invade epithelial cells. However, both genes are inactivated in *Y. pestis* (Parkhill et al., 2001), which no longer colonizes and proliferates in the gut. Other important pseudogenes in *Y. pestis* such as *ureD*, *rcsA*, and *pde*, the pseudogenization of which is necessary for the flea-host-flea cycle of *Y. pestis* (Sebbane et al., 2001; Sun et al., 2008; Chen et al., 2010). Furthermore, a frameshift mutation in *Y. pestis rcsD* might compensate for the fitness cost imposed by loss of *rcsA* function and preserve flea-mammal plague transmission cycles (Guo et al., 2023). Some other genome reductions, affecting the survival of *Y. pestis* in its ambient conditions, are likely driven by natural selection.

Overall, our data suggest that the deletion of YPO0623 may confer fitness advantages to *Y. pestis* in its survival under natural environmental pressures. On one hand, the upregulation of T6SS-A cluster resulting from the deletion of YPO0623 likely enhances *Y. pestis* resistance to various environmental stresses, creating a scenario where nonsense mutations in the gene become fixed within the genome. On the other hand, considering the low aspartate aminotransferase activity, the loss of YPO0623 may reduce the fitness cost for *Y. pestis*, thereby promoting the growth of the bacterium. Notably, a recent study found that YPO0623 (referred as y3555 in strain KIM10+), in conjunction with other genes, plays a crucial role in optimizing aromatic amino acid metabolism and is induced under conditions of hyperosmotic salinity stress. It might contribute to the survival of fleas for *Y. pestis* (Martinez-Chavarria et al., 2020). We have to acknowledge that the tale of YPO0623 is far more complex than we expected. More detailed investigations are needed to fully uncover the function and evolution of YPO0623.

## Data availability statement

The datasets presented in this study can be found in online repositories. The names of the repository/repositories and accession number(s) can be found below: The data has been released on NMDC with the corrected accession numbers NMDC40041575-NMDC40041608 (https://nmdc.cn/resource/genomics/sra/detail/NMDC40041575).

## Ethics statement

The animal study was approved by Beijing Institute of Microbiology and Epidemiology. The study was conducted in accordance with the local legislation and institutional requirements.

## Author contributions

JJ: Writing – original draft. LX: Writing – original draft. YW: Data curation, Writing – original draft. ZS: Methodology, Writing – original draft. ZX: Data curation, Writing – original draft. YL: Data curation, Writing – original draft. YZ: Writing – original draft. WY: Writing – original draft. LS: Writing – original draft. YC: Writing – original draft. YT: Writing – review & editing. YH: Writing – review & editing. ZD: Writing – review & editing. YC: Writing – review & editing. RY: Writing – review & editing. KS: Writing – review & editing. YS: Writing – review & editing.

## Supplementary material

Supplementary Figure 1 SDS-PAGE of purified protein. Lane 1 is bacterial supernatant, Lane 2 is supernatant after ultrasound, Lane 3 is 10 mM imidazole eluent, Lane 4 is 20 mM imidazole eluent, Lane 5 is 40 mM imidazole eluent, Lane 6 is 250 mM imidazole eluent, and Lane 7 is eluent after desalination column.

Supplementary Figure 2 Standard curve of NADH-Na_2_ at different pH. **(A)** pH=2.0. **(B)** pH=3.0. **(C)** pH=6.0. **(D)** pH=7.0. **(E)** pH=8.0. **(F)** pH=9.0.

Supplementary Figure 3 Comparative transcriptomics of strain 201-WT and 201-Δ0623 cultured at different temperatures. **(A)** Differential gene expression of 201-WT and 201-Δ0623 at 26°C. **(B)** Differential gene expression of 201-WT and 201-Δ0623 at 37°C. **(C)** Correlations among the expression levels of 25 genes measured with RNA-seq and qRT-PCR were analyzed using linear regression.

Supplementary Figure 4 KEGG enrichment analysis comparing the RNA-seq data between 201-Δ0623 and 201-WT. **(A)** Up-regulated KEGG enrichment analysis at 26°C. **(B)** Down-regulated KEGG enrichment analysis at 26°C. **(C)** Up-regulated KEGG enrichment analysis at 37°C. **(D)** Down-regulated KEGG enrichment analysis at 37°C.
