# Supplementary figures and images for: Characterization of an aspartate aminotransferase encoded by YPO0623 with frequent nonsense mutations in Yersinia pestis
Source: Front Cell Infect Microbiol. 2023 Nov 28;13:1288371. doi: 10.3389/fcimb.2023.1288371 (PMC10713766; doi:10.3389/fcimb.2023.1288371)

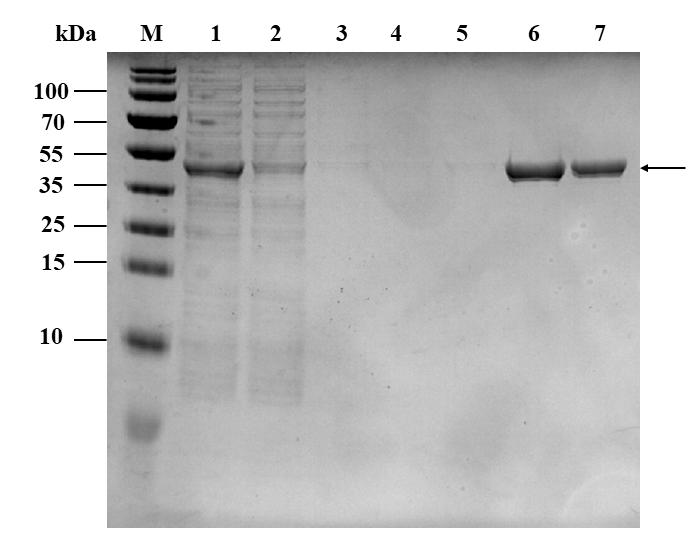

Supplement: Supplementary Figure 1 — SDS-PAGE of purified protein. Lane 1 is bacterial supernatant, Lane 2 is supernatant after ultrasound, Lane 3 is 10 mM imidazole eluent, Lane 4 is 20 mM imidazole eluent, Lane 5 is 40 mM imidazole eluent, Lane 6 is 250 mM imidazole eluent, and Lane 7 is eluent after desalination column. [file Image_1.tif]

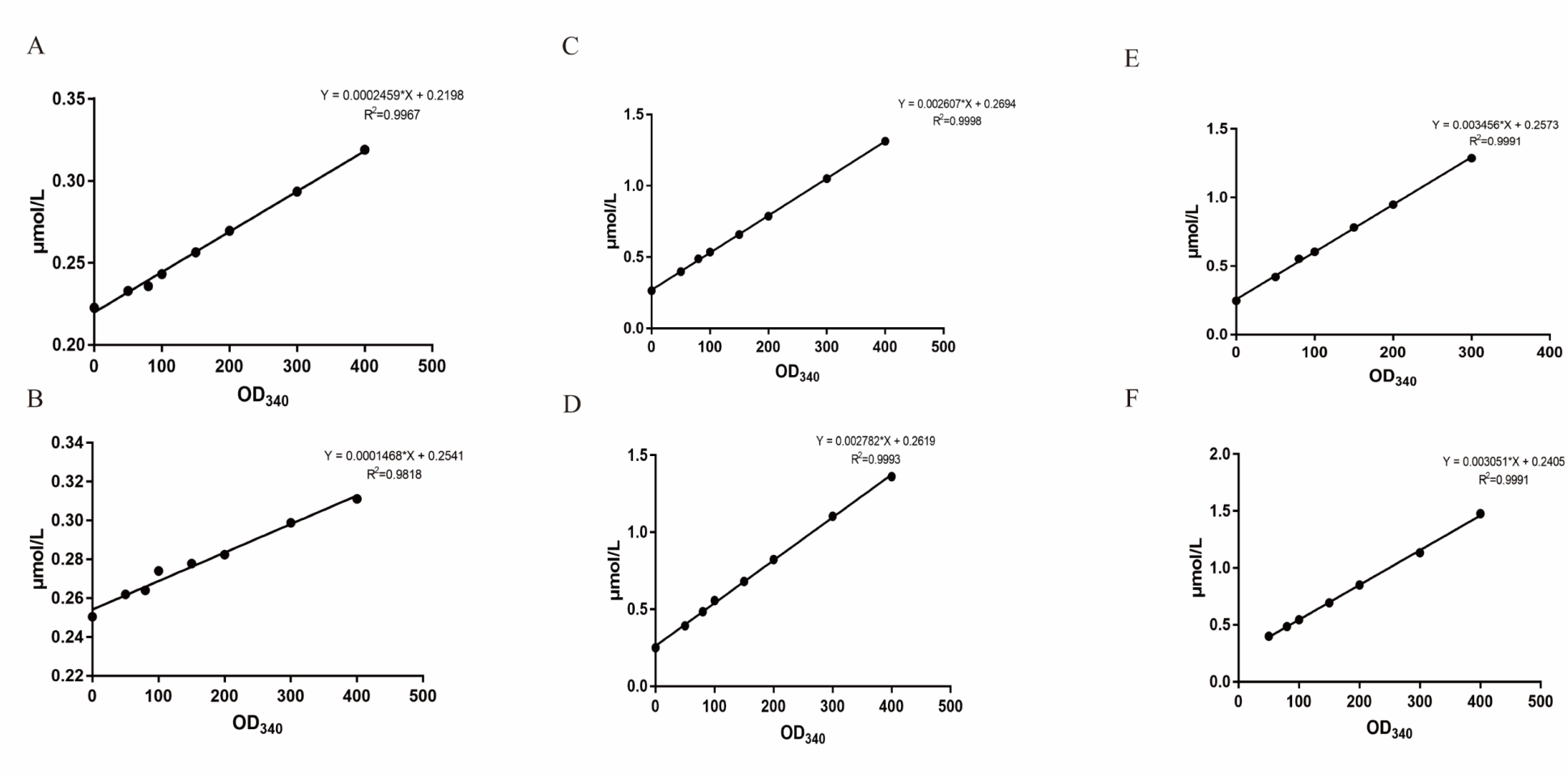

Supplement: Supplementary Figure 2 — Standard curve of NADH-Na2 at different pH. (A) pH=2.0. (B) pH=3.0. (C) pH=6.0. (D) pH=7.0. (E) pH=8.0. (F) pH=9.0. [file Image_2.tif]

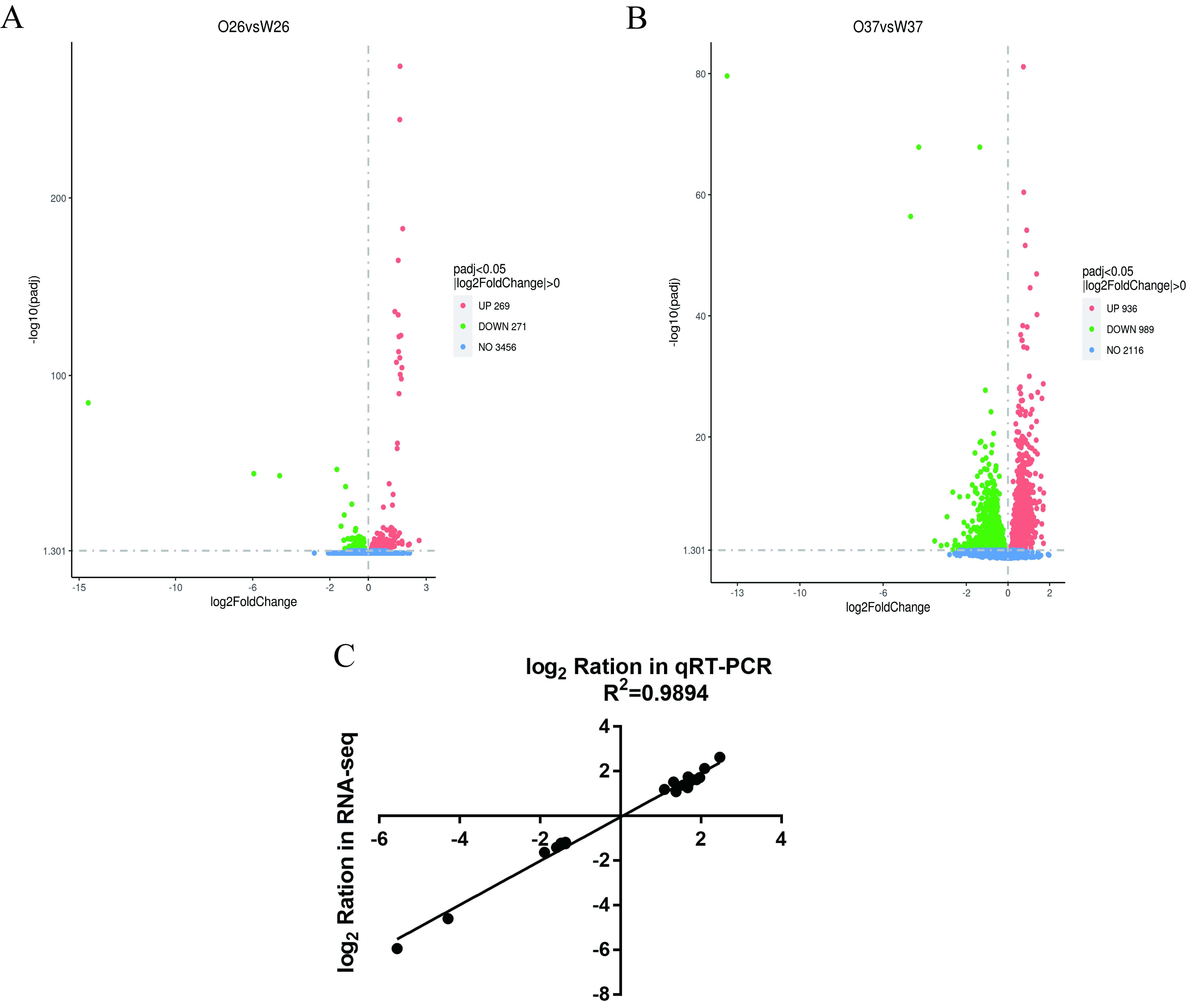

Supplement: Supplementary Figure 3 — Comparative transcriptomics of strain 201-WT and 201-Δ0623 cultured at different temperatures. (A) Differential gene expression of 201-WT and 201-Δ0623 at 26°C. (B) Differential gene expression of 201-WT and 201-Δ0623 at 37°C. (C) Correlations among the expression levels of 25 genes measured with RNA-seq and qRT-PCR were analyzed using linear regression. [file Image_3.tif]

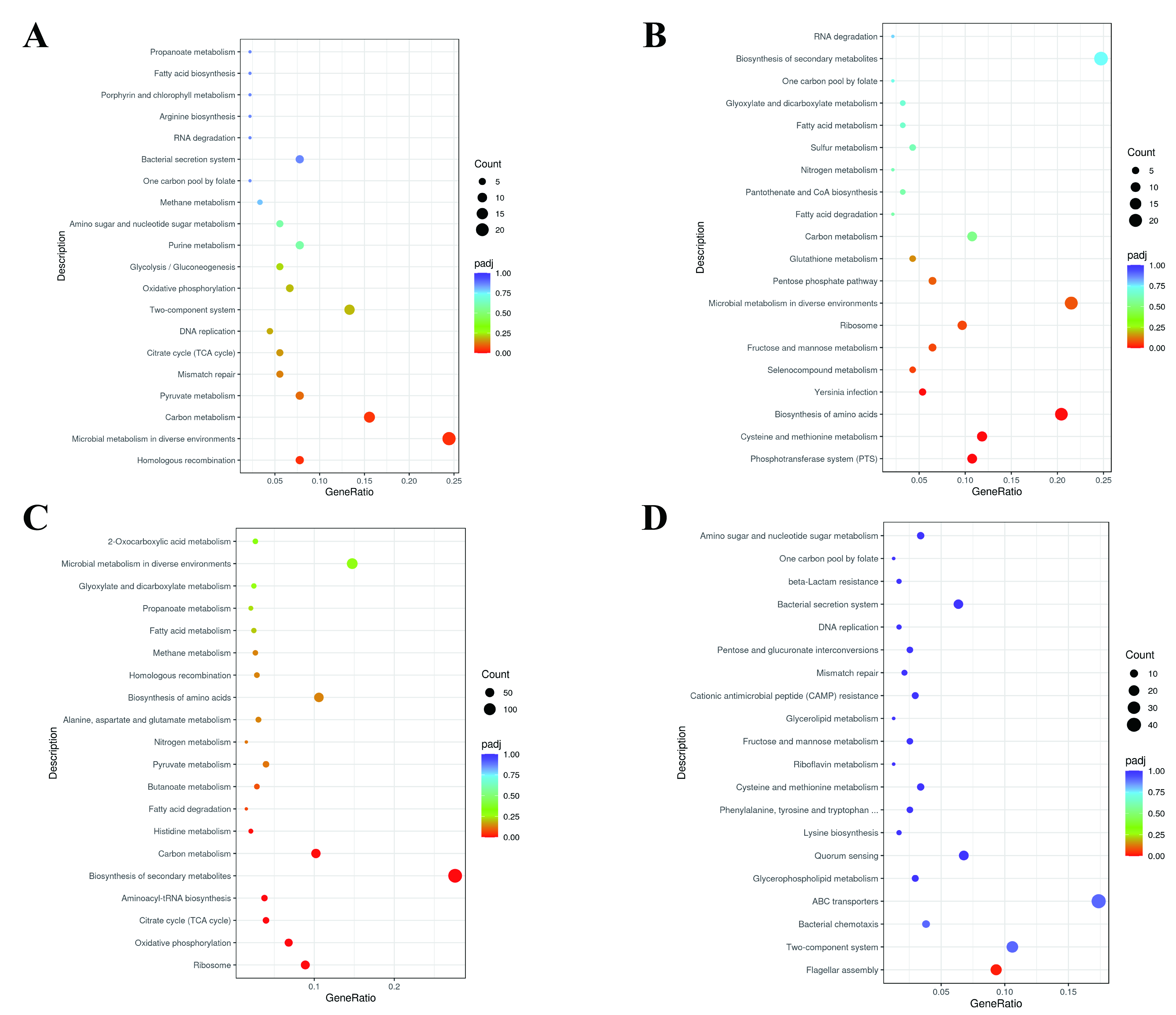

Supplement: Supplementary Figure 4 — KEGG enrichment analysis comparing the RNA-seq data between 201-Δ0623 and 201-WT. (A) Up-regulated KEGG enrichment analysis at 26°C. (B) Down-regulated KEGG enrichment analysis at 26°C. (C) Up-regulated KEGG enrichment analysis at 37°C. (D) Down-regulated KEGG enrichment analysis at 37°C. [file Image_4.tif]
