# Supplementary material for: Characterization of an aspartate aminotransferase encoded by YPO0623 with frequent nonsense mutations in Yersinia pestis
Source: Front Cell Infect Microbiol. 2023 Nov 28;13:1288371. doi: 10.3389/fcimb.2023.1288371 (PMC10713766; doi:10.3389/fcimb.2023.1288371)
Supplement: Supplementary file 5 [file Table_1.docx]

Supplementary Table 1 Bacterial strains and plasmids used in this study.

| Strain or plasmid | Genotype | Ref or source |
| --- | --- | --- |
| *E. coli*  S17λpir | Tpr Smr recA thi pro hsdR−M+ (RP4-2-Tc::Mu: KanrTn7) λpir | [1] |
| S17-pDS132-YPO0623 | pDS132-YPO0623 was introduced into S17λpir | This study |
| DH-5α | *sup*E44 ∆*lac*U169 (*φ80lac*Z∆M15) *hsd*R17 *rec*A1 *end*A1 *gyr*A96 *thi*-1 *rel*A1 | [2] |
| DH-5α- pET-28 (a+)-YPO0623 | pET-28 (a+)-YPO0623 was introduced into DH-5α | This study |
| BL21 (ED3) | F^−^ *ompT gal dcm lon hsdS_B_* (r_B_^−^ m_B_^−^) λDE3 [*lacI lacUV*5 -T7 gene *1 ind1 sam7 nin5*] | [2] |
| BL21- pET-28 (a+)-YPO0623 | pET-28 (a+)-YPO0623 was introduced into BL21 | This study |
| ***Yersinia pestis*** |  |  |
| strain 201 | *Yersinia pestis* biovar Microtus strain 201, WT | [2] |
| 201-Δ0623 | deleted YPO0623 based on strain 201 | This study |
| 201-Δ0623-Comp | 201-Δ0623 containing plasmid pACYC184-YPO0623 | This study |
| **plasmids** |  |  |
| pDS132 | Suicide vector, Derived from pCVD442, without IS1 sequences. *bla* gene replaced by the cat gene | [3] |
| pACYC184 | Cloning vector, Cmr Tetr | [4] |
| pDS132-YPO0623 | homology arm of YPO0623 were inserted into pDS132 | This study |
| pACYC184-YPO0623 | YPO0623 gene was inserted into pACYC184, used for the complement of YPO0623 | This study |
| pET-28 (a+) | Amplifing the kanamycin resistance cassette, Kan^R^ | YouBio, China |
| pET-28 (a+)-YPO0623 | YPO0623 gene was inserted into pET-28 (a+), used for the expression of YPO0623 protein | This study |

Supplementary Table 2 Primers used in the gene knockout, complement, protein expression and qRT-PCR.

| Primer | Primer sequence (5’-3’) |
| --- | --- |
| 0623-F | ATCATCGAT**AAGCTT**AAATTCCGCTGTTAGAG |
| 0623-R | CGGCGTAGA**GGATCC**TTATCCATGGCAGATCCCCT |
| pACYC184-F | CGGTTCAAAGAGTTGGT |
| pACYC184-R | TGATGTCGGCGATATAG |
| Pre0623-F | AGAGGTACC**GCATGC**TCGTAAATAGCTGAAGTCAC |
| Pre0623-R | CGCGAAAAATGAGTCCTTCAGGTATTCCCCTTTAATAAAT |
| Post0623-F | TGAAGGACTCATTTTTCGCG |
| Post0623-R | TTCCCGGGA**GAGCTC**TGTGGTATCCGATATTGGGG |
| pDS132-F | GTTTCTGTTGCATGGGCATAAAG |
| pDS132-R | AACAAGCCAGGGATGTAACG |
| Inter-0623-F | TAAAATCACCGAAAAAACCA |
| Inter-0623-R | TTGACCATAAGGAACACCTG |
| BamH Ⅰ-0623-F | *CGC***GGATCC**ATGAATACGATTAGAAGAAAAATGCG |
| Hind Ⅲ-0623-R | *CCC***AAGCTT**TTATCCATGGCAGATCCCC |
| T7 -F | TAATACGACTCACTATAGGG |
| T7-Ter -R | GCTAGTTATTGCTCAGCGG |
| *hslU*-F | CGAAGTGGGTTATGTTG |
| *hslU*-R | AGAAGGCTCCTGTGATT |
| *hslV*-F | AAGTCCGCCGTCTTTAT |
| *hslV*-R | CACGTCACCGTTACCAG |
| *clpB*-F | TCATTAGATATGGGGTC |
| *clpB*-R | GTATTCATCGAGGGTAG |
| *htpG*-F  *htpG*-R  *tssE*-F  *tssE*-R  *tssC*-F  *tssC*-R  *tssB*-F  *tssB*-R  *tssK*-F  *tssK*-R  *tssJ*-F  *tssJ*-R  *tssH*-F  *tssH*-R  *tssG*-F  *tssG*-R  *tssF*-F  *tssF*-R  *tssC*-F  *tssC*-R  *tssB*-F  *tssB*-R  *ibpB*-F  *ibpB*-R  *ibpA*-F  *ibpA*-R | AGAAATCTCTGACGACG  CGAATCTATCAAACCAC  TAATTGGGATCGAGGGA  AATCAGATGAGGTGGCG  GCCCCTGATTTCTTTGG  CGGCTGACATCCTCGTA  TGATGGGGAAGAAGACA  GTAGACGAGAGCGGAAA  AACTTCCTGCCACCCAT  CCGATAACCCACCGACT  CTCTCATCAATCCTGGG  TGACGACTTCACCTCCT  ACAAAGGGTGGATGGAA  CGCAAGATGATGAGAGG  CTGTATTTCTCGGTTCC  TGGGTGAGGTCTGTATG  GCTTTCTTGAGTTCCCG  TGTATTTTCAGCGTGCG  GGTTGGTGTTCAAGGAT  GTGGCAAAAATGTAAGG  TCCTCAAATGGAAAAAC  AACATCATCATCCTGGC  CTGACGATAACCACTAT  GAGAACTCTTTACGAAC  GCTCCATTGTATCGTTC  CGGTAGTTATTTTCGTC |
| *glnK*-F | ATGGACGAGAAAAAAGA |
| *glnK*-R | CTGCCACGAAACAGATA |
| YPO0625-F | AATCAATGTTTCTGTGG |
| YPO0625-R | GATAATTTTTTGGGTCT |
| YPO0624-F | ATCGGTAGGAACGCTAT |
| YPO0624-R | ATGCTTGTAAGTTTGGG |
| YPO0627-F | TAATGGGATTCTGTATG |
| YPO0627-R | TTTATCTTTTGTTGTTC |
| YPO0628-F | CCGAGACGATGTACCCC |
| YPO0628-R | TCCTGCCCCCCAACTAA |
| YPO3862-F | CTTGCCTGATAGAGTTG |
| YPO3862-R | TGTTGATAGCGAGATTC |
| YPO0626-F | TTGCGATGCTCTGTATG |
| YPO0626-R | CACGAGGGTAGTTCTTT |
| PF-F | CGAGAAGCGGATGTACT |
| PF-R | GTGTAGCCCAGAAGAAA |
| *tcaA1*-F | AGATACCGAAGCACACG |
| *tcaA1*-R | TATTCTCCGCTAACAGG |

Black body-enzyme cleavage site; Underline-homologous sequence on the plasmid; Italics-protective base.

1. Brem, D., et al., *Functional analysis of yersiniabactin transport genes of Yersinia enterocolitica.* Microbiology, 2001. **147**(Pt 5): p. 1115-27.

2. Song, Y., et al., *Complete genome sequence of Yersinia pestis strain 91001, an isolate avirulent to humans.* DNA Res, 2004. **11**(3): p. 179-97.

3. Philippe, N., et al., *Improvement of pCVD442, a suicide plasmid for gene allele exchange in bacteria.* Plasmid, 2004. **51**(3): p. 246-55.

4. Chang, A.C. and S.N. Cohen, *Construction and characterization of amplifiable multicopy DNA cloning vehicles derived from the P15A cryptic miniplasmid.* J Bacteriol, 1978. **134**(3): p. 1141-56.
